# Vortical Differential Scattering of Spatio‐Temporal Optical Vortex With Arbitrarily Oriented Orbital Angular Momentum by Chiral Spherical Particle

**DOI:** 10.1002/nap2.70246

**Published:** 2026-08-11

**Authors:** Yiyu Shi, Zhiwei Cui, Zhanfei Liu, Shiguo Chen, Xinxing Zhou

**Affiliations:** ^1^ School of Physics Xidian University Xi'an China; ^2^ Key Laboratory of Low‐Dimensional Quantum Structures and Quantum Control of Ministry of Education School of Physics and Electronics Hunan Normal University Changsha China; ^3^ Key Laboratory of Physics and Devices in Post‐Moore Era College of Hunan Province Changsha China; ^4^ Hunan Research Center of the Basic Discipline for Quantum Effects and Quantum Technologies Hunan Normal University Changsha China

**Keywords:** arbitrarily oriented orbital angular momentum (OAM), polychromatic GLMT, spatiotemporal optical vortex (STOV), vortical differential scattering

## Abstract

We theoretically investigate the vortical differential scattering (VDS) of spatiotemporal optical vortex (STOV) carrying arbitrary orbital angular momentum (OAM) orientation by chiral particles within the framework of polychromatic generalized Lorenz–Mie theory (GLMT). Numerical results show that STOVs with a nonzero longitudinal OAM component exhibit significant vortical dichroism (VD), whereas those with purely transverse OAM do not. Mechanistically, transverse OAM's zero net optical chirality density and collapsed spatial energy flow vortex lack the requisite torque and asymmetric multipolar excitations to drive chiral responses. Furthermore, it is found that the larger topological charges and tighter spatiotemporal scales significantly enhance the VD. Our work establishes a solid theoretical foundation for future explorations of the chiral dynamics of STOV, paving the way for applications in enantiomer sensing, advanced chiral spectroscopy, and precise optical manipulation.

## Graphical abstract

The vortical differential scattering of spatiotemporal optical vortices by chiral particles is investigated. It is revealed that a longitudinal orbital angular momentum component is essential for inducing vortical dichroism, whereas a purely transverse OAM yields no chiral response. Enhanced dichroism is achieved with larger topological charges and tighter spatiotemporal confinement, offering new insights for chiral sensing and manipulation.

## 1 Introduction

Chirality, fundamentally defined as a geometric property where an object cannot be superimposed onto its mirror image due to spatial symmetry breaking, is a ubiquitous phenomenon in nature from fundamental particles [1] to macroscopic molecules [2]. Analogous to matter chirality, light fields also exhibit chiral properties [3, 4, 5, 6, 7]. The most classical representative is circularly polarized light (CPL), for which the handedness originates from the helical trajectory of the rotating electric field vector. The differential interaction between CPL and chiral matter gives rise to circular dichroism (CD) [8, 9, 10, 11, 12], which has been extensively utilized as a primary spectroscopic tool for chiral enantiomer sensing. Beyond the realm of spin angular momentum associated with polarization, recent advancements in structured light have illuminated that optical vortex beams carrying orbital angular momentum (OAM) also possess inherent macroscopic chirality [13, 14, 15, 16]. This specific form of optical chirality is governed by the handedness of the helical phase wavefront, which originates from to the OAM. Consequently, the differential scattering or absorption of an optical vortex with opposite OAM by chiral particles manifests as vortical dichroism (VD) [17, 18, 19, 20]. As an effective complementary method to conventional CD, VD can generate a stronger signal by employing a large OAM value, without the need for circular polarization. Therefore, it has attracted intensive research interest for its wide applications in probing chiral light–matter interactions, advanced chiral spectroscopy, and optical manipulation [21].

Generally, the OAM of spatial vortex beams is strictly longitudinal, meaning it is oriented in the direction of beam propagation [22, 23, 24]. In 2012, Bliokh et al. proposed a space‐time generalization of the known spatial vortex beam, now known as the spatiotemporal optical vortex (STOV), carrying OAM at an arbitrary angle relative to the mean momentum [25]. Following this pioneering work, the experimental evidence of STOVs with controllable transverse OAM was presented by Chong et al. through the application of a spiral phase in the spatial‐frequency domain [26]. Such STOVs are potentially applicable in ultra‐capacity information carriers [27], high‐dimensional data encoding [28], edge detection spatiotemporal differentiator [29], Second‐harmonic generation [30], attosecond electron sheet [31], and so on. Subsequently, STOVs with arbitrary OAM orientations have been successfully realized experimentally [32, 33, 34]. Such STOVs with arbitrarily oriented OAM provide more degrees of freedom to control and exhibit more abundant features. Das et al. developed a quantum theory of OAM fluctuations for STOVs with an arbitrarily oriented OAM, extending beyond the paraxial limit [35]. Tang et al. analyzed the centroid shifts of STOVs with arbitrarily oriented OAM upon planar reflection and refraction [36]. Despite these significant research advances, the intrinsic structural asymmetries of STOV and its interplay with chiral matter remain an unexplored frontier. Unlike traditional spatial vortex beams, STOVs feature a tunable OAM vector in three‐dimensional space. This orientational degree of freedom may introduce complex, spatiotemporally coupled field gradients that interact with the 3D architectures of chiral microparticles in a highly directional chiroptical manner. This leads to a highly intriguing and fundamental question: when these STOVs possessing arbitrary OAM orientations interact with chiral spherical particles, can they induce the VD? If so, what are the underlying mechanisms and physical principles governing this chiroptical response?

To address these questions, this paper investigates the vortical differential scattering of STOVs carrying arbitrary OAM orientations by chiral particles. Based on the polychromatic GLMT, we derive the beam shape coefficients (BSCs) of STOVs. We calculate the VDS characteristics of STOVs by chiral particles for different OAM orientations, and analyze the physical mechanisms from the perspectives of local optical chirality density and Poynting vector distribution. Finally, we explore the effects of topological charge and spatiotemporal scale on the VDS characteristics of STOVs. Our findings not only provide meaningful insights into chiral light–matter interactions but also establish a robust theoretical basis for designing advanced chiroptical sensing and precise optical manipulation technologies.

## 2 Theory

As shown in Figure 1, the angular spectrum representation of the STOV carrying OAM with an orientation angle (Θ,Φ) can be described by the LG function with radial index zero and topological charge *l* as

(1) $$\tilde{\psi }\left(k\right)=w_{0}^{\left|l\right|}{\left[\begin{array}{l} \cos\;\Theta \;\cos\;\Phi k_{x}+\cos\;\Theta \;\sin\;\Phi k_{y}-\sin\;\Theta \left(k_{z}-k_{0}\right)\\ +i\text{sgn}\left(l\right)\left(\cos\;\Phi k_{y}-\sin\;\Phi k_{x}\right) \end{array}\right]}^{\left|l\right|}\exp\left[-\frac{w_{0}^{2}}{4}\left(k_{x}^{2}+k_{y}^{2}+{\left(k_{z}-k_{0}\right)}^{2}\right)\right],$$

where $w_{0}$ is the spatiotemporal scale and $k_{0}$ is the central wave number. Based on the relationship $\left(k_{x},k_{y},k_{z}\right)=k\left(\sin\;\alpha \;\cos\;\beta ,\sin\;\alpha \;\sin\;\beta ,\cos\;\alpha \right)$, the profile of an STOV in spherical coordinates (r,θ,ϕ) can be calculated by the inverse Fourier transform as

(2) $$\psi \left(r,t\right)=\frac{1}{2}\int_{-\infty }^{+\infty }k^{2}\int_{0}^{\pi /2}\int_{0}^{2\pi }\tilde{\psi }\left(k,\alpha ,\beta \right)\sin\;2\;\alpha d\alpha d\beta \;\exp\left(ik\cdot r-ikct\right)dk,$$

with exp(ik·r)=exp{ikr[sinαsinθcos(ϕ−β)+cosαcosθ]} and light velocity *c*. For $\Theta =\Phi =0^{\circ }$, the STOV degenerates into a standard Laguerre–Gaussian (LG) pulse with purely longitudinal OAM and the phase fronts exhibit a continuous helical twist along the azimuthal direction. When $\Theta =\Phi =30^{\circ }$, the OAM possesses both longitudinal and transverse projections, giving rise to strong spatiotemporal coupling. When $\Theta =\Phi =90^{\circ }$, the OAM becomes purely transverse and the singularity line completely detaches from the propagation axis and rotates into the spatiotemporal plane.

**FIGURE 1:**
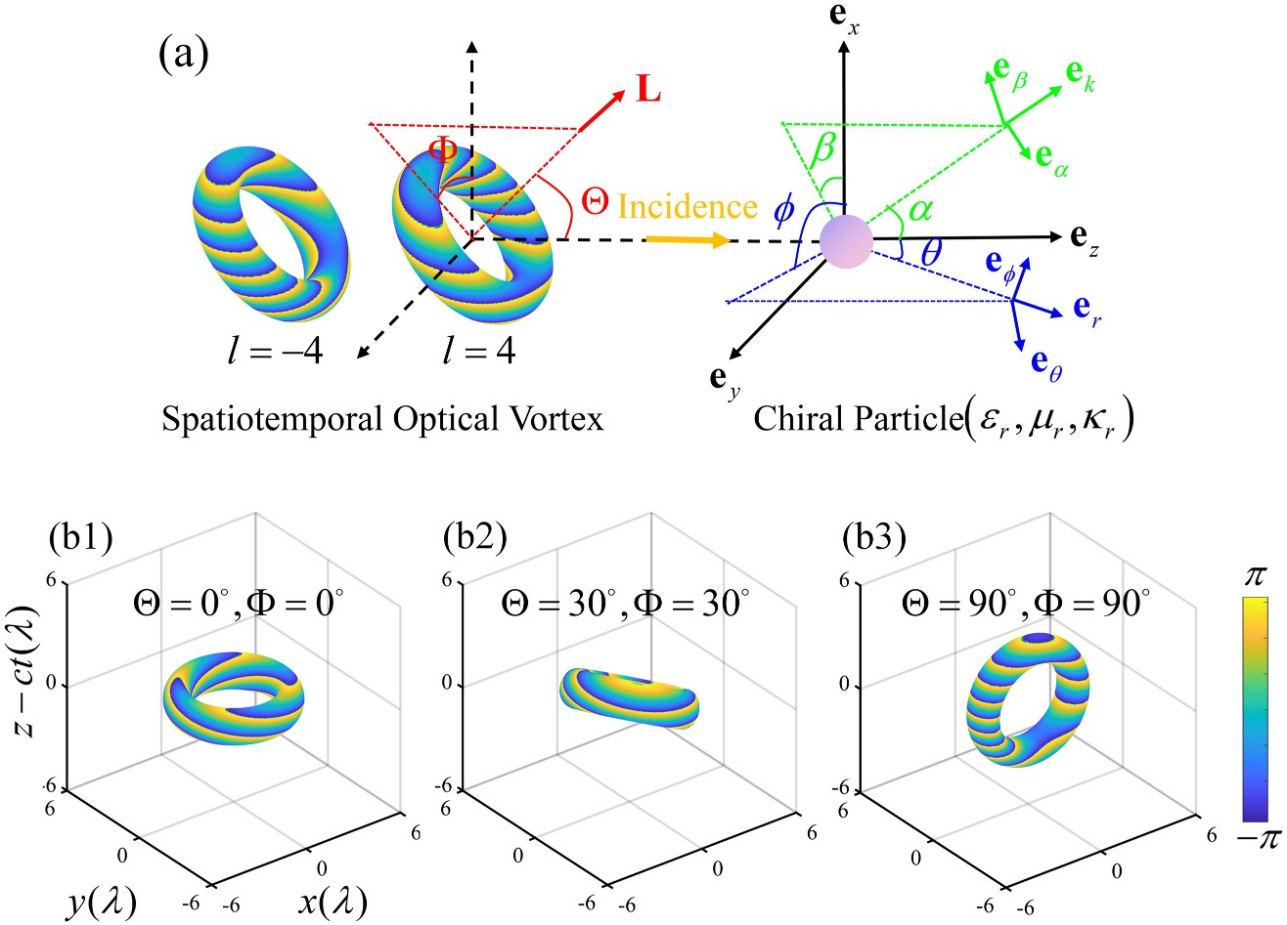
(a) Schematic of the chiral spherical particle illuminated by STOV with orientation angle (Θ,Φ) of OAM. The (k,α,β) and (r,θ,ϕ) are the spherical coordinates for wave vector k and location vector r, respectively, with the α,θ and β,ϕ being polar and azimuthal angles, respectively. (b1)–(b3) 3D wave packet of STOV for differently oriented OAM.

Furthermore, assuming that the incident STOV with polarization state $\left(p_{x},p_{y}\right)$ propagates along the positive z‐axis in the global coordinate system (x,y,z), we can introduce two mutually orthogonal vector potential functions as follows:

(3) $$A=\left(p_{x}e_{x}+p_{y}e_{y}\right)\psi ,A^{⊥}=\left(p_{x}e_{y}-p_{y}e_{x}\right)\psi .$$

Under the quasi‐monochromatic condition $\left|\partial \psi /\partial t\right|\ll k_{0}c\left|\psi \right|$, the electric and magnetic fields of the STOV in medium with permittivity ε and permeability μ can be expressed in terms of A and $A^{⊥}$ as follows:

(4) $$\begin{array}{ll} E^{\text{inc}} & =\left[A+k_{0}^{-2}\nabla \left(\nabla \cdot A\right)+ik_{0}^{-1}\nabla \times A^{⊥}\right]/2,\\ H^{\text{inc}} & =\sqrt{\frac{\varepsilon }{\mu }}\left[A^{⊥}+k_{0}^{-2}\nabla \left(\nabla \cdot A^{⊥}\right)-ik_{0}^{-1}\nabla \times A\right]/2. \end{array}$$

Substituting Equation (3) into Equation (4), then into Equation (5), we obtain

(5) $$E^{\text{inc}}=\frac{1}{4}\int_{-\infty }^{+\infty }k^{2}\int_{0}^{\pi /2}\int_{0}^{2\pi }\tilde{\psi }\left(k,\alpha ,\beta \right)E_{\text{pw}}\;\exp\left(ik\cdot r-ikct\right)\sin\;2\;\alpha d\alpha d\beta dk,$$

(6) $$H^{\text{inc}}=\frac{1}{4}\sqrt{\frac{\varepsilon }{\mu }}\int_{-\infty }^{+\infty }k^{2}\int_{0}^{\pi /2}\int_{0}^{2\pi }\hat{\psi }\left(k,\alpha ,\beta \right)H_{\text{pw}}\;\exp\left(ik\cdot r-ikct\right)\sin\;2\;\alpha d\alpha d\beta dk,$$

in which

(7) $$E_{\text{pw}}=2\;\cos^{2}\frac{\alpha }{2}\left[e_{\alpha }\left(p_{x}\;\cos\;\beta +p_{y}\;\sin\;\beta \right)-e_{\beta }\left(p_{x}\;\sin\;\beta -p_{y}\;\cos\;\beta \right)\right],$$

(8) $$H_{\text{pw}}=2\;\cos^{2}\frac{\alpha }{2}\left[e_{\beta }\left(p_{x}\;\cos\;\beta +p_{y}\;\sin\;\beta \right)+e_{\alpha }\left(p_{x}\;\sin\;\beta -p_{y}\;\cos\;\beta \right)\right],$$

and $e_{\alpha }$ and $e_{\beta }$ are basis vectors in the spherical angular spectrum momentum space (k,α,β).

For investigating the interaction between light and a spherical particle, the GLMT is an ideal choice due to its accuracy and efficiency. Notably, the conventional GLMT is applicable only to monochromatic waves. For an STOV composed of polychromatic waves, its electric and magnetic fields can be obtained in the framework of the polychromatic GLMT as follows:

(9) $$E^{\text{inc}}=\int_{\infty }{\tilde{E}}^{\text{inc}}\exp\left(-ikct\right)dk=\int_{\infty }\sum_{n=1}^{\infty }\sum_{m=-n}^{n}c_{n}^{pw}\left[ia_{mn}\left(k\right)N_{mn}^{\left(1\right)}\left(kr\right)-b_{mn}\left(k\right)M_{mn}^{\left(1\right)}\left(kr\right)\right]\exp\left(-ikct\right)dk,$$

(10) $$H^{\text{inc}}=\int_{\infty }{\tilde{H}}^{\text{inc}}\exp\left(-ikct\right)dk=\int_{\infty }\sqrt{\frac{\varepsilon }{\mu }}\sum_{n=1}^{\infty }\sum_{m=-n}^{n}c_{n}^{pw}\left[a_{mn}\left(k\right)M_{mn}^{\left(1\right)}\left(kr\right)+ib_{mn}\left(k\right)N_{mn}^{\left(1\right)}\left(kr\right)\right]\exp\left(-ikct\right)dk,$$

where $c_{n}^{pw}=i^{n}\left(n+0.5\right)/\left[n\left(n+1\right)\right]$ is the plane wave factor, $a_{mn}\left(k\right)$ and $b_{mn}\left(k\right)$ are the so‐called beam shape coefficients (BSCs), and $M_{mn}^{\left(1\right)}$ and $N_{mn}^{\left(1\right)}$ are the first kind of vector spherical wave functions (VSWFs) that are defined by

(11) $$M_{mn}^{\left(1\right)}\left(kr\right)=j_{n}\left(kr\right)\left[im{\tilde{\pi }}_{n}^{m}\left(\theta \right)e_{\theta }-{\tilde{\tau }}_{n}^{m}\left(\theta \right)e_{\phi }\right]\exp\left(im\phi \right),$$

(12) $$N_{mn}^{\left(1\right)}\left(kr\right)=\frac{1}{kr}\left\{n\left(n+1\right)j_{n}\left(kr\right){\tilde{P}}_{n}^{m}\left(\theta \right)e_{r}+{\left[krj_{n}\left(kr\right)\right]}^{\prime }\left[{\tilde{\tau }}_{n}^{m}\left(\theta \right)e_{\theta }+im{\tilde{\pi }}_{n}^{m}\left(\theta \right)e_{\phi }\right]\right\}\exp\left(im\phi \right),$$

where $e_{r}$, $e_{\theta }$, and $e_{\phi }$ are the unit vectors in spherical coordinates (r,θ,ϕ), $j_{n}\left(\cdot \right)$ is the spherical Bessel function, and ${\tilde{\pi }}_{n}^{m}\left(\theta \right)={\tilde{P}}_{n}^{m}\left(\cos\;\theta \right)/\sin\;\theta$ and ${\tilde{\tau }}_{n}^{m}\left(\theta \right)=d{\tilde{P}}_{n}^{m}\left(\cos\;\theta \right)/d\theta$ are the angular functions with ${\tilde{P}}_{n}^{m}\left(\cdot \right)$ being the normalized associated Legendre function. It is well established that monochromatic plane waves can also be expanded in terms of an infinite series of VSWFs as follows:

(13) $$E_{\text{pw}}\;\exp\left(ik\cdot r\right)=\sum_{n=1}^{\infty }\sum_{m=-n}^{n}c_{n}^{pw}\left[ia_{mn}^{\prime }N_{mn}^{\left(1\right)}\left(kr\right)-b_{mn}^{\prime }M_{mn}^{\left(1\right)}\left(kr\right)\right],$$

(14) $$H_{\text{pw}}\;\exp\left(ik\cdot r\right)=\sqrt{\frac{\varepsilon }{\mu }}\sum_{n=1}^{\infty }\sum_{m=-n}^{n}c_{n}^{pw}\left[a_{mn}^{\prime }M_{mn}^{\left(1\right)}\left(kr\right)+ib_{mn}^{\prime }N_{mn}^{\left(1\right)}\left(kr\right)\right],$$

where the expansion coefficients $a_{mn}^{\prime }$ and $b_{mn}^{\prime }$ are given by

(15) $$\left(\begin{array}{l} a_{mn}^{\prime }\\ ib_{mn}^{\prime } \end{array}\right)=-\cos^{2}\frac{\alpha }{2}\cdot \frac{4\;\exp\left(-im\beta \right)}{2n+1}\left\{\begin{array}{l} \left[m{\tilde{\pi }}_{n}^{m}\left(\alpha \right)+{\tilde{\tau }}_{n}^{m}\left(\alpha \right)\right]\exp\left(i\beta \right)\\ \mp \left[m{\tilde{\pi }}_{n}^{m}\left(\alpha \right)-{\tilde{\tau }}_{n}^{m}\left(\alpha \right)\right]\exp\left(-i\beta \right) \end{array}\right\}.$$

Substituting Equations (13) and (14) into Equations (5) and (6) and comparing with Equations (9) and (10), it is finally derived that

(16) $$\begin{array}{cc} \left(\begin{array}{l} a_{mn}\left(k\right)\\ ib_{mn}\left(k\right) \end{array}\right)= & \frac{k^{2}}{4}\int_{0}^{\pi /2}\int_{0}^{2\pi }\tilde{\psi }\left(k,\alpha ,\beta \right)\left(\begin{array}{l} a_{mn}^{\prime }\\ ib_{mn}^{\prime } \end{array}\right)\sin\;2\;\alpha d\alpha d\beta \\ & =-\frac{k^{2+\left|l\right|}}{\left(2n+1\right)}\int_{0}^{\pi /2}\int_{0}^{2\pi }d\alpha d\beta \times \cos^{2}\frac{\alpha }{2}\exp\left(-im\beta \right)\sin\;2\;\alpha \\ & \times \left[\begin{array}{l} F_{nm}^{1}\left(\alpha \right)\exp\left(i\beta \right)-F_{nm}^{2}\left(\alpha \right)\exp\left(-i\beta \right)\\ F_{nm}^{1}\left(\alpha \right)\exp\left(i\beta \right)+F_{nm}^{2}\left(\alpha \right)\exp\left(-i\beta \right) \end{array}\right]\\ & \times {\left[\left(\begin{array}{l} \cos\;\Theta \;\cos\;\Phi \;\sin\;\alpha \;\cos\;\beta \\ +\cos\;\Theta \;\sin\;\Phi \;\sin\;\alpha \;\sin\;\beta \\ -\sin\;\Theta \left(k_{z}-k_{0}\right) \end{array}\right)+i\text{sgn}\left(l\right)\left(\begin{array}{l} -\sin\;\Phi \;\sin\;\alpha \;\cos\;\beta \\ +\cos\;\Phi \;\sin\;\alpha \;\sin\;\beta \end{array}\right)\right]}^{\left|l\right|} \end{array}$$

where $F_{nm}^{1}=\left[m{\tilde{\pi }}_{n}^{m}\left(\alpha \right)+{\tilde{\tau }}_{n}^{m}\left(\alpha \right)\right]$ and $F_{nm}^{2}=i\left[m{\tilde{\pi }}_{n}^{m}\left(\alpha \right)-{\tilde{\tau }}_{n}^{m}\left(\alpha \right)\right]$.

When the STOV is scattered by a spherical particle with refractive index $n_{p}$, radius $r_{p}$, and chiral parameter κ, the scattered electric and magnetic fields can be expressed as follows:

(17) $$E^{\text{sca}}=\int_{\infty }{\tilde{E}}^{\text{sca}}\exp\left(-ikct\right)dk=\int_{\infty }\sum_{n=1}^{\infty }\sum_{m=-n}^{n}c_{n}^{pw}\left[ia_{mn}^{s}\left(k\right)N_{mn}^{\left(1\right)}\left(kr\right)-b_{mn}^{s}\left(k\right)M_{mn}^{\left(1\right)}\left(kr\right)\right]\exp\left(-ikct\right)dk,$$

(18) $$H^{\text{sca}}=\int_{\infty }{\tilde{H}}^{\text{sca}}\exp\left(-ikct\right)dk=\int_{\infty }\sqrt{\frac{\varepsilon }{\mu }}\sum_{n=1}^{\infty }\sum_{m=-n}^{n}c_{n}^{pw}\left[a_{mn}^{s}\left(k\right)M_{mn}^{\left(1\right)}\left(kr\right)+ib_{mn}^{s}\left(k\right)N_{mn}^{\left(1\right)}\left(kr\right)\right]\exp\left(-ikct\right)dk,$$

where, $M_{mn}^{\left(3\right)}$ and $N_{mn}^{\left(3\right)}$ are the third‐kind VSWFs, which are similar to $M_{mn}^{\left(1\right)}$ and $N_{mn}^{\left(1\right)}$ except the spherical Bessel function $j_{n}\left(kr\right)$ being replaced by the Hankel function of the first kind $h_{n}^{\left(1\right)}\left(kr\right)$. Specially, the BSCs $a_{mn}^{s}\left(k\right)=d_{n}\left(k\right)a_{mn}\left(k\right)+ie_{n}\left(k\right)b_{mn}\left(k\right)$ and $b_{mn}^{s}\left(k\right)=c_{n}\left(k\right)b_{mn}\left(k\right)-ie_{n}\left(k\right)b_{mn}\left(k\right)$, in which

(19) $$\begin{array}{cc} c_{n}\left(k\right) & =\left[A_{n}^{\left(2\right)}V_{n}^{\left(1\right)}+A_{n}^{\left(1\right)}V_{n}^{\left(2\right)}\right]Q_{n},\\ d_{n}\left(k\right) & =\left[B_{n}^{\left(1\right)}W_{n}^{\left(2\right)}+B_{n}^{\left(2\right)}W_{n}^{\left(1\right)}\right]Q_{n},\\ e_{n}\left(k\right) & =\left[A_{n}^{\left(1\right)}W_{n}^{\left(2\right)}-A_{n}^{\left(2\right)}W_{n}^{\left(1\right)}\right]Q_{n}, \end{array}$$

(20) $$A_{n}^{\left(j\right)}=D_{n}^{\left(1\right)}\left(x_{j}\right)/n_{r}-D_{n}^{\left(1\right)}\left(x_{0}\right),B_{n}^{\left(j\right)}=D_{n}^{\left(1\right)}\left(x_{j}\right)-D_{n}^{\left(1\right)}\left(x_{0}\right)/n_{r},$$

(21) $$W_{n}^{\left(j\right)}=D_{n}^{\left(1\right)}\left(x_{j}\right)/n_{r}-D_{n}^{\left(3\right)}\left(x_{0}\right),V_{n}^{\left(j\right)}=D_{n}^{\left(1\right)}\left(x_{j}\right)-D_{n}^{\left(3\right)}\left(x_{0}\right)/n_{r},$$

(22) $$D_{n}^{\left(1\right)}\left(x\right)={\left[xj_{n}\left(x\right)\right]}^{\prime }/\left[xj_{n}\left(x\right)\right],D_{n}^{\left(3\right)}\left(x\right)={\left[xh_{n}^{\left(1\right)}\left(x\right)\right]}^{\prime }/\left[xh_{n}^{\left(1\right)}\left(x\right)\right],$$

(23) $$Q_{n}=\frac{j_{n}\left(x_{0}\right)/h_{n}^{\left(1\right)}\left(x_{0}\right)}{V_{n}^{\left(1\right)}W_{n}^{\left(2\right)}+V_{n}^{\left(2\right)}W_{n}^{\left(1\right)}},$$

where $x_{0}=kr_{p}$, $x_{1}=k_{1}r_{p}$, $x_{2}=k_{2}r_{p}$, $k_{1}=k\left(n_{r}+\kappa_{r}\right)$, and $k_{2}=k\left(n_{r}-\kappa_{r}\right)$, with $r_{p}$, $n_{p}$, and κ being the radius, refractive index, and chirality parameter of the particle, respectively. In the far‐zone r→∞, $M_{mn}^{\left(3\right)}$ and $N_{mn}^{\left(3\right)}$ can be approximated as follows:

(24) $${Μ}_{mn}^{\left(3\right)}\left(kr\right)\approx \frac{{\left(-i\right)}^{n}\exp\left(ikr\right)}{kr}\left[m\pi_{n}^{m}\left(\theta \right)e_{\theta }+i\tau_{n}^{m}\left(\theta \right)e_{\phi }\right]\exp\left(im\phi \right),$$

(25) $$N_{mn}^{\left(3\right)}\left(kr\right)\approx \frac{{\left(-i\right)}^{n}\exp\left(ikr\right)}{kr}\left[\frac{n\left(n+1\right)}{ikr}{\tilde{P}}_{n}^{m}\left(\theta \right)e_{r}+im\pi_{n}^{m}\left(\theta \right)e_{\theta }+\tau_{n}^{m}\left(\theta \right)e_{\phi }\right]\exp\left(im\phi \right).$$

In Eqs. (17) and (18), the radial component $E_{r}^{\text{sca}}$ of the scattered electric field can be neglected, and monochromatic components $E_{\phi }^{\text{sca}}$ and $E_{\theta }^{\text{sca}}$ can be approximated as follows:

(26) $$\left[\begin{array}{l} E_{\phi }^{\text{far}-\text{sca}}\left(k\right)\\ E_{\theta }^{\text{far}-\text{sca}}\left(k\right) \end{array}\right]\approx \frac{i\;\exp\left(ikr\right)}{kr}\left[\begin{array}{l} -S_{1}\left(k\right)\\ S_{2}\left(k\right) \end{array}\right],$$

where

(27) $$S_{1}\left(k\right)=\sum_{n=1}^{\infty }\sum_{m=-n}^{n}c_{n}^{pw}\left[a_{mn}^{s}\left(k\right)\tau_{n}^{m}\left(\theta \right)-imb_{mn}^{s}\left(k\right)\pi_{n}^{m}\left(\theta \right)\right]\exp\left(im\phi \right),$$

(28) $$S_{2}\left(k\right)=\sum_{n=1}^{\infty }\sum_{m=-n}^{n}c_{n}^{pw}\left[ma_{mn}^{s}\left(k\right)\pi_{n}^{m}\left(\theta \right)-ib_{mn}^{s}\left(k\right)\tau_{n}^{m}\left(\theta \right)\right]\exp\left(im\phi \right).$$

The scattering intensity $I_{\text{sca}}$ of the far‐zone scattered electric field is defined as follows:

(29) $$I_{\text{sca}}=\frac{1}{k_{0}^{2}}\int_{-\infty }^{+\infty }\left[{\left|S_{1}\left(k\right)\right|}^{2}+{\left|S_{2}\left(k\right)\right|}^{2}\right]dk,$$

and the VD is defined by a dissymmetry factor as follows:

(30) $$\text{VD}=\frac{I_{\text{sca}}^{l}-I_{\text{sca}}^{-l}}{I_{\text{sca}}^{l}+I_{\text{sca}}^{-l}}\times 2,$$

where $I_{\text{sca}}^{l}$ and $I_{\text{sca}}^{-l}$ are scattering intensities of the STOV with opposite topological charge.

Furthermore, it is important to consider the influence of material dispersion in practical applications. For narrow‐band pulses ($\Delta k/k_{0}\ll 1$) or within transparent windows, material parameters remain approximately constant, aligning with our central‐frequency calculations. However, for ultrashort broadband pulses interacting near strong electronic resonances, the frequency‐dependent refractive index $n_{p}\left(k\right)$ and chiral parameter $\kappa_{r}\left(k\right)$ will modulate the multipolar Mie coefficients across the pulse spectrum, selectively amplifying resonant differential scattering. Because our polychromatic GLMT is formulated as a frequency‐domain integral, incorporating empirical dispersion simply requires substituting constant parameters with functions $n_{p}\left(k\right)$ and $\kappa_{r}\left(k\right)$ in Equations (19), (20), (21), (22), (23).

## 3 Results

In this section, we first numerically simulate the reconstructed STOV and compare it with the original counterpart to verify the validity of the BSCs. Then, we calculate the $I_{\text{sca}}^{l}$, $I_{\text{sca}}^{-l}$, and VD of STOV for different OAM orientations. The underlying physical mechanism behind these phenomena is analyzed from the perspectives of local optical chirality density and transverse distribution of Poynting vectors. Finally, the influences of the topological charge *l* and spatiotemporal scale $w_{0}$ on the backward scattering intensity and VD are depicted. In the following simulations, if not otherwise specified, it is assumed that the wavelength, orientation angle, topological charge, and spatiotemporal scale of the STOV are λ=0.6328μm, $\left(\Theta =30^{\circ },\Phi =30^{\circ }\right)$, l=4, and $w_{0}=2.0\lambda$, respectively; the relative refractive index, chiral parameter, radius of particle are $n_{p}=2.0$, $\kappa_{r}=0.1$, and $r_{p}=0.6328\;\mu m$, respectively.

First, we can verify the validity of BSCs for STOV from Figure 2 based on the comparison between the original STOV calculated by Equation (2) and its reconstructed field calculated by Equations (9) and (10). This comparison is fundamentally crucial for validating the mathematical rigorousness of our theoretical framework before investigating the scattering dynamics. When the orientation angles of OAM are set to $\Theta =30^{\circ }$ and $\Phi =30^{\circ }$, the spatiotemporal distribution of the STOV is symmetric along an axis inclined at exactly $30^{\circ }$ as shown in Figure 2a,b. Notably, the reconstructed field shows slight discrepancies from the original field in Figure 2c, which stem from the inherent physical assumptions underlying their respective mathematical descriptions. The analytical formulation used to construct the original incident STOV is conventionally derived under the paraxial approximation, which inevitably introduces slight inaccuracies when describing tightly localized or strongly focused spatiotemporal fields. In contrast, the reconstructed field synthesized through the VSWF superposition rigorously satisfies the exact Maxwell equations. It should be noted that this subtle difference will not affect the overall trend calculated later. The other method to verify the validity of our theoretical framework from the perspective of asymptotic degeneration can be seen in Supporting Information S1.

**FIGURE 2:**
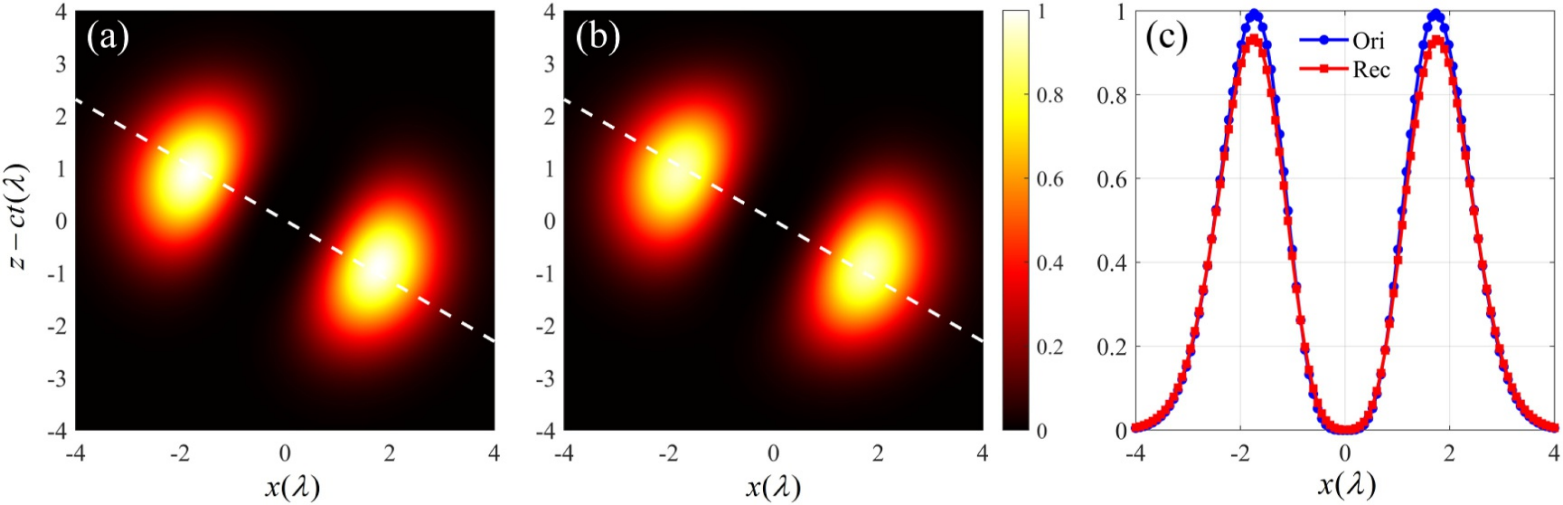
Validity verification of the BSCs for an STOV with orientation angle $\left(\Theta =30^{\circ },\Phi =30^{\circ }\right)$. (a)The original field of the STOV. (b)The reconstructed field of the STOV. (c) Comparison between the original and reconstructed STOVs along the white dashed line in panels (a) and (b).

Figure 3 illustrates the scattering intensities and the corresponding VD of STOV interacting with a chiral spherical particle under varying OAM orientations. Notably, to eliminate the interference of extrinsic chirality, we use unpolarized illumination and azimuthal observation angle compensation (see more details in Supporting Information S1). For the canonical case $\left(\Theta =0^{\circ },\Phi =0^{\circ }\right)$ shown in Figure 3a1,a2, the scattering fields excited by incident beams with opposite topological charges exhibit substantial deviations, particularly at scattering angles exceeding $90^{\circ }$. In this case, the calculated relative VD remarkably surpasses 100%. However, when both orientation angles are dynamically increased to $30^{\circ }$, as plotted in Figure 3b1,b2, the maximum VD attenuates to approximately 50%. Interestingly, despite this quantitative decrease in VD, the maximum absolute intensity of VDS is enhanced to roughly twice that of the conventional LG pulse. Ultimately, Figure 3c1,c2 illustrate that when the OAM orientation angles become $\left(\Theta =90^{\circ },\Phi =90^{\circ }\right)$ with the STOV degenerating into a state carrying purely transverse OAM, the VD response completely vanishes. These results unveil a crucial physical trade‐off regarding chiral light–matter interactions. For a Mie sphere whose radius is considerably smaller than the spatiotemporal scale $w_{0}$ of the STOV, the LG pulse (longitudinal OAM) yields the most pronounced relative VD. Nevertheless, due to its inherent hollow energy distribution at the beam axis, the overall scattering field intensity remains exceedingly weak. By intentionally tilting the OAM vector, the absolute intensity of VDS can be effectively amplified. This unique property significantly improves the resolution and detectability of the differential signals, demonstrating that utilizing tilted spatiotemporal vortices constitutes a highly advantageous strategy for the robust sensing of chiral Mie particles.

**FIGURE 3:**
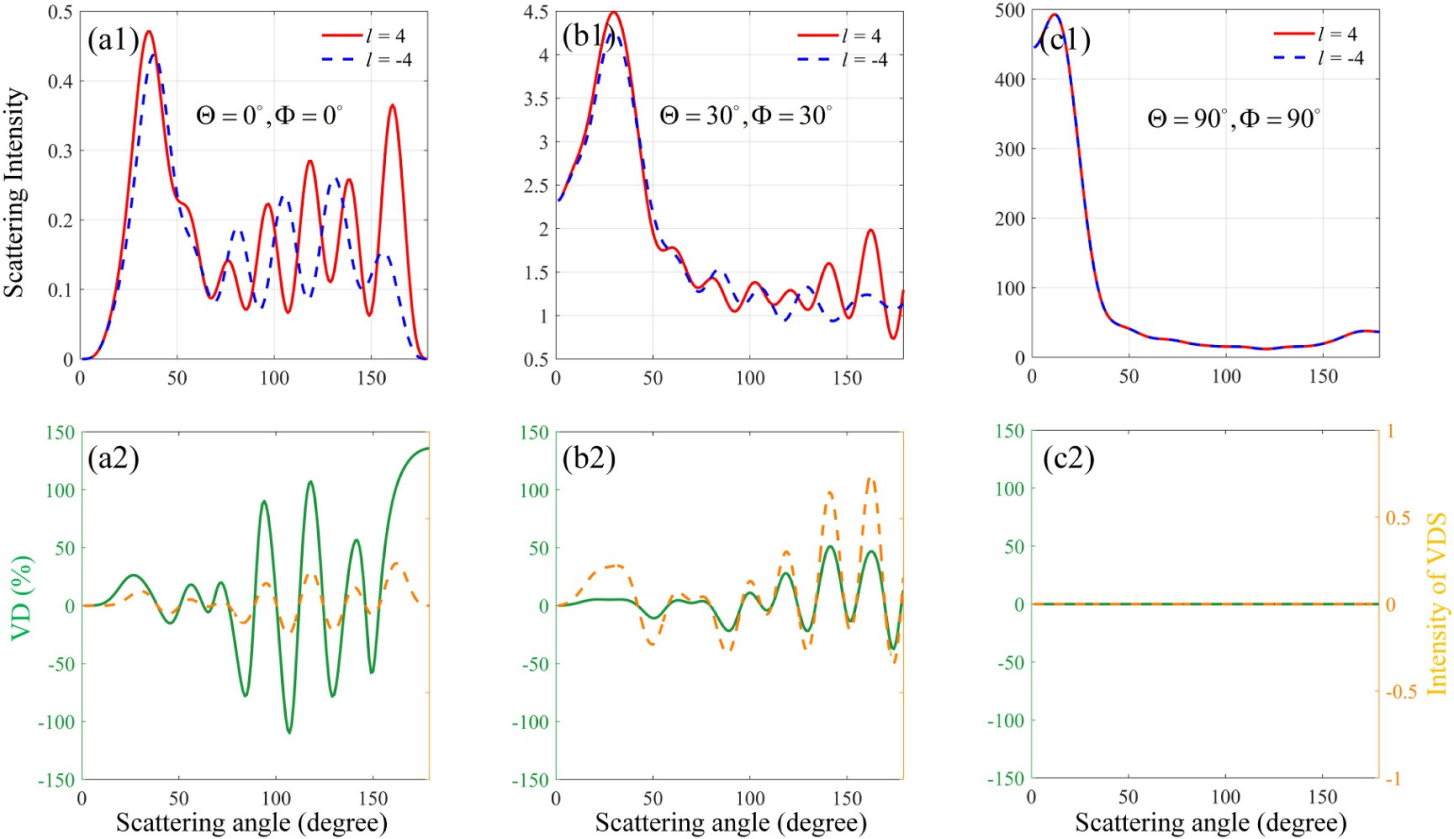
Intensity of VDS and corresponding VD for an STOV with different OAM orientation angles. (a) $\Theta =0^{\circ },\Phi =0^{\circ }$. (b) $\Theta =30^{\circ },\Phi =30^{\circ }$. (c) $\Theta =90^{\circ },\Phi =90^{\circ }$.

To illustrate the physical mechanisms behind the VDS above, the local optical chirality density at the central frequency is plotted in Figure 4. We focus our analysis on the central wave number component, which provides an intuitive geometric visualization and maintains physical universality. Optical chirality density acts as the primary electromagnetic excitation source for driving chiral light–matter interactions. As demonstrated in Figure 4a,b, when the OAM vector possesses a longitudinal component, the spatial integration of the total optical chirality density across the *xoy* plane yields a nonzero value. In contrast, when the STOV carries purely transverse OAM as shown in Figure 4c, a nonzero local optical chirality density still emerges due to complex spatiotemporal coupling effects, yet it exhibits an exact spatial antisymmetry. This distribution pattern results in a net zero integrated chirality density over the *xoy* plane. Fundamentally, the magnitude of the VDS from a Mie particle is governed jointly by the spatial integral of the optical chirality density for incident STOV and the high‐order multipolar resonance effects (see more details in Supporting Information S1). Consequently, when this integrated optical chirality density vanishes for the case of purely transverse OAM $\left(\Theta =90^{\circ },\Phi =90^{\circ }\right)$, the asymmetric excitation sources for all multipole orders are equal to zero, leading to the complete disappearance of the VDS.

**FIGURE 4:**
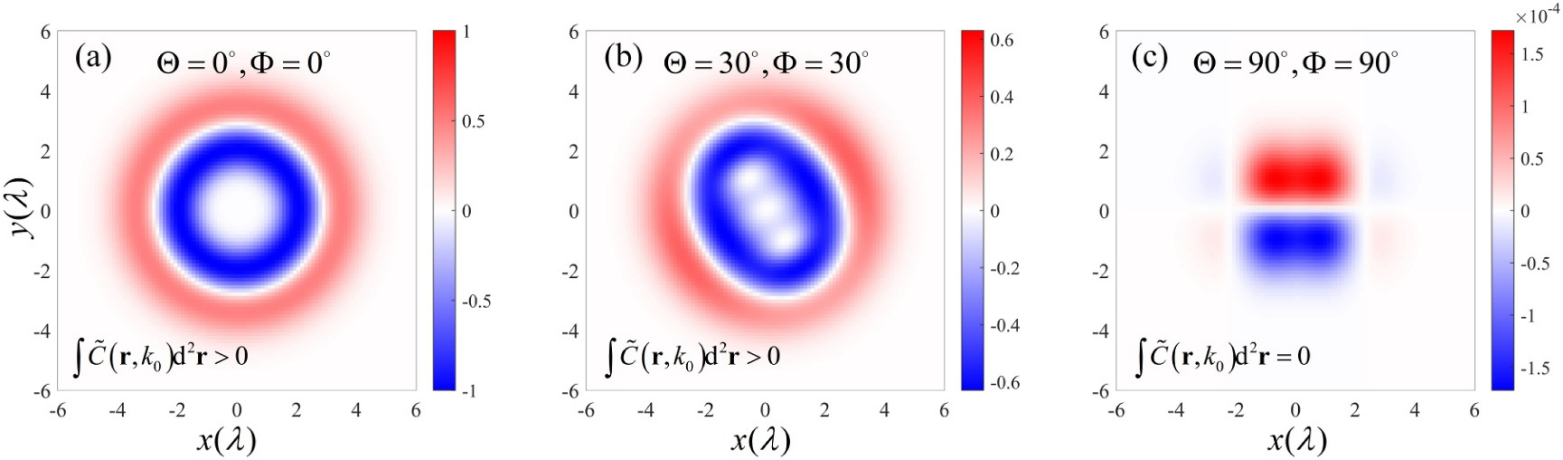
Center wave number $k_{0}$ component of local optical chirality density for an STOV with different OAM orientation angles. (a) $\Theta =0^{\circ },\Phi =0^{\circ }$. (b) $\Theta =30^{\circ },\Phi =30^{\circ }$. (c) $\Theta =90^{\circ },\Phi =90^{\circ }$.

Aside from local optical chirality density, the physical mechanisms can be understood from the electromagnetic energy transport by examining the spatial streamlines of the Poynting vector as shown in Figure 5. When the OAM retains a longitudinal component, as presented in Figure 5a,b, the optical field inherently supports a local azimuthal energy flow that continuously rotates around the propagation axis. This transverse spatial circulation of energy interacts differentially with the intrinsic structural handedness of the chiral particle, giving rise to constructive or destructive interference mechanism that ultimately generates the robust VDS. Conversely, when the OAM is strictly constrained to a purely transverse orientation, as presented in Figure 5c, the spatial topological phase singularity degenerates. As a consequence, the previously circulating spatial energy streamlines completely collapse into planar and symmetric linear trajectories. Without the spatial azimuthal circulation of energy, the optical field lacks the requisite topological torque needed to drive an asymmetric electrodynamic response within the chiral particle (see more details in Supporting Information S1).

**FIGURE 5:**
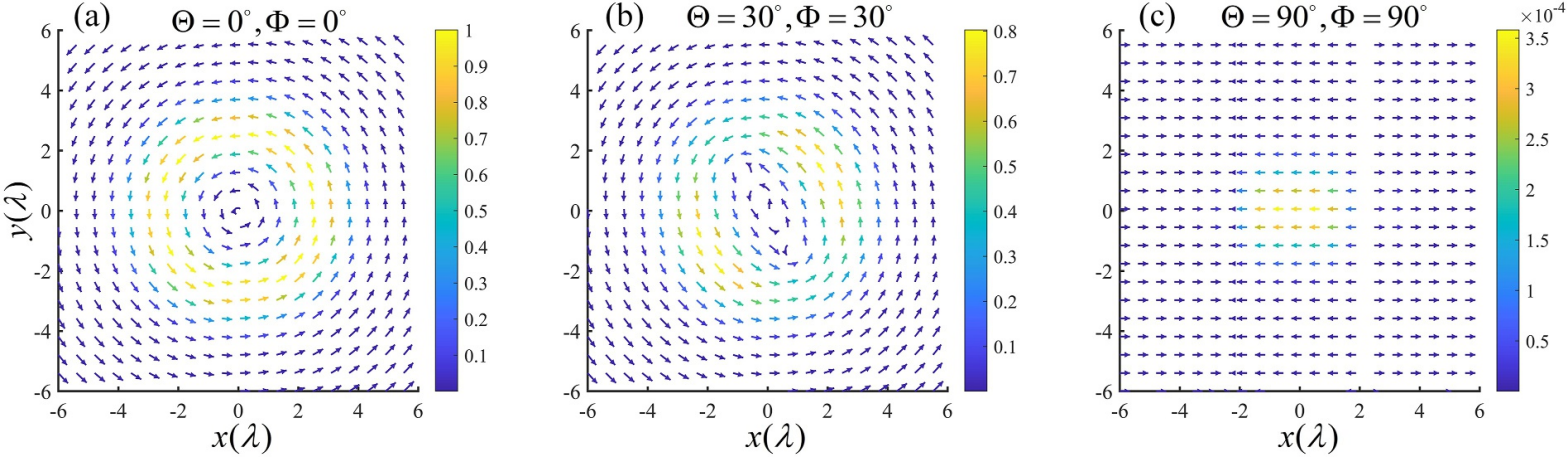
Central wave number $k_{0}$ component of Poynting vector distribution for an STOV with different OAM orientation angles. (a) $\Theta =0^{\circ },\Phi =0^{\circ }$. (b) $\Theta =30^{\circ },\Phi =30^{\circ }$. (c) $\Theta =90^{\circ },\Phi =90^{\circ }$.

Finally, Figure 6 depicts the influences of the STOV's topological charge *l* and spatiotemporal scale $w_{0}$ on the VDS and VD. As deduced from the angular distributions in Figure 3, the chiral response is most pronounced in the backward scattering regime spanning from $150^{\circ }$ to $180^{\circ }$. Considering the practical convenience of signal collection in experimental optical setups, we define the total scattered energy integrated over this solid angle as the backward‐cone integrated scattering intensity. The numerical evaluations reveal distinct evolutionary trends. As the absolute value of the topological charge *l* increases, the total reflected intensity exhibits a nonmonotonic behavior, initially decreasing before subsequently increasing. Conversely, expanding the spatiotemporal scale $w_{0}$ leads to a gradual attenuation of the backward‐cone integrated VDS intensity. More importantly, the corresponding VD demonstrates a significant and robust enhancement when either the topological charge *l* is increased or the spatiotemporal scale $w_{0}$ is decreased. Physically, the enhancement of VD stems from amplified electrodynamic interactions. On the one hand, a larger topological charge *l* produces a sharper azimuthal phase gradient. This intensifies both the macroscopic topological torque and the local optical chirality density, driving a more robust asymmetric response from the chiral particle. On the other hand, reducing the spatiotemporal scale $w_{0}$ increases field confinement, which drastically amplifies the spatial field gradients of the STOV. Since chiral light–matter interactions driven by high‐order multipoles strongly depend on spatial field gradients, this tighter field confinement significantly enhances the asymmetric excitation, ultimately enhancing the VD. Notably, these fundamental scaling laws are universally valid for both enantiomers with opposite handedness, offering vital theoretical paradigms for optimizing the optical sensing and sorting of chiral materials.

**FIGURE 6:**
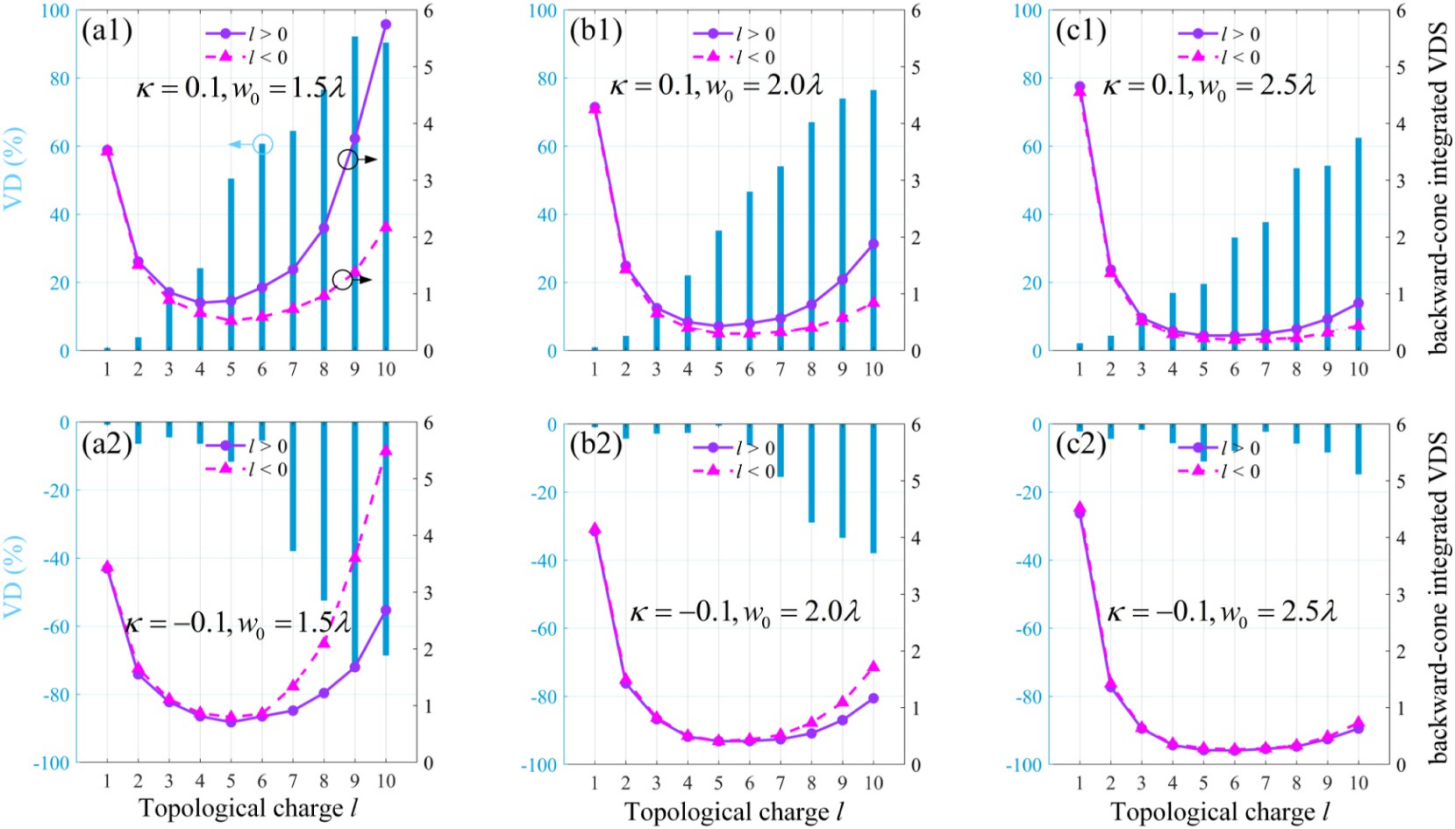
Backward‐cone integrated VDS of an STOV with orientation angle $\left(\Theta =30^{\circ },\Phi =30^{\circ }\right)$ and corresponding VD for different topological charge *l*, spatiotemporal scale $w_{0}$, and chiral parameter $\kappa_{r}$. (a1)–(c1) $\kappa_{r}=0.1$. (a2)–(c2) $\kappa_{r}=-0.1$.

Before concluding, we briefly discuss the experimental feasibility. STOVs with controllable OAM orientations can be generated using a 4‐*f* pulse shaper with a programmable spatial light modulator (SLM) or by using astigmatic mode converters. To achieve unpolarized illumination without degrading the phase singularities, two orthogonally polarized pulses can be combined with a subcoherence time delay or modulated via a rapid electro‐optic switcher. Regarding detection, our backward‐cone integrated strategy (Figure 6) avoids complex angular tracking by deploying a high‐numerical‐aperture objective to collect the total backscattered light (150°–180°). When reversing the topological charge via the SLM, the detector directly measures the integrated power difference, dramatically relaxing optomechanical alignment tolerances and improving the signal‐to‐noise ratio.

## 4 Conclusion

In conclusion, this study systematically explores the VDS of STOVs with arbitrarily oriented OAM scattered by chiral particles. Using a polychromatic GLMT, we derive the BSCs for STOVs and numerically evaluate their scattering fields from a chiral Mie sphere. Numerical results reveal that the orientation of the OAM profoundly affects both the scattering intensity and the VD. As the OAM transitions from a longitudinal to a transverse orientation, the scattering intensity increases, yet the dichroism correspondingly weakens. In particular, purely transverse OAM fails to excite VD. The underlying mechanism is ascribed to the zero net optical chirality density of STOV with transverse OAM, which precludes asymmetric multipolar excitations. Alternatively, for purely transverse OAM, the spatial energy flow vortex of transverse OAM collapses, lacking the requisite torque to drive asymmetric chiral responses. Furthermore, we have established that VD in the backward scattering regime is significantly enhanced when employing larger topological charges or tighter spatiotemporal scales. This study provides a valuable theoretical framework for exploring the chiral dynamics of STOVs and may find applications in the development of enantiomer sensing, chiral spectroscopy, and optical manipulation technologies.

## Author Contributions

**Yiyu Shi:** writing – original draft, writing – review and editing, data curation. **Zhiwei Cui:** conceptualization, visualization. **Zhanfei Liu:** formal analysis, investigation. **Shiguo Chen:** project administration, software. **Xinxing Zhou:** supervision.

## Funding

This study is funded by the National Natural Science Foundation of China (92464205, 12374273, and 12421005), Shaanxi Fundamental Science Research Project for Mathematics and Physics (25JSY021), and Hunan Provincial Major Sci‐Tech Program (2023ZJ1010).

## Conflicts of Interest

The authors declare no conflicts of interest.

## Supporting information


Supporting Information S1

## Data Availability

Data underlying the results presented in this paper are not publicly available at this time but may be obtained from the authors upon reasonable request.
