# Supplementary material for: Vortical Differential Scattering of Spatio‐Temporal Optical Vortex With Arbitrarily Oriented Orbital Angular Momentum by Chiral Spherical Particle
Source: Nanophotonics. 2026 Aug 11;15(15):e70246. doi: 10.1002/nap2.70246 (PMC13642570; doi:10.1002/nap2.70246)
Supplement: Supplementary file 1 [file NAP2-15-e70246-s001.docx]

**Vortical differential scattering of spatio- temporal optical vortex with arbitrarily oriented orbital angular momentum by chiral spherical particle: supplemental document**

Yiyu Shi,^1^ Zhiwei Cui,^1,5^ Zhanfei Liu,^1^ Shiguo Chen,^1^ and Xinxing Zhou ^2,3,4,6^

*^1^School of Physics, Xidian University, Xi’an, 710071, China*

*^2^Key Laboratory of Low-Dimensional Quantum Structures and Quantum Control of Ministry of Education, School of Physics and Electronics, Hunan Normal University, Changsha 410081, China*

*^3^Key Laboratory of Physics and Devices in Post-Moore Era, College of Hunan Province, Changsha 410081, China*

*^4^Hunan Research Center of the Basic Discipline for Quantum Effects and Quantum Technologies, Hunan Normal University, Changsha 410081, China*

*^5^[zwcui@mail.xidian.edu.cn](mailto:22201215120@stu.xidian..edu.cn)*

*^6^xinxingzhou@hunnu.edu.cn*

**S1. Analytical Proof of Asymptotic Degeneration from Polychromatic STOV to Monochromatic LG Beam**

To demonstrate the self-consistency and mathematical rigor of our polychromatic GLMT for the STOV, it is essential to perform an asymptotic degeneration analysis. In this section, we analytically prove that under specific limiting conditions, the BSCs of the polychromatic STOV smoothly and precisely degenerate into the BSCs of a monochromatic LG beam.

In our theoretical framework, the STOV is composed of continuous frequency components. We can decouple the global spatiotemporal scale$w_{0}$ into a transverse spatial waist radius$w_{s}$ and a longitudinal temporal pulse length$w_{t}$. Considering the canonical longitudinal orientation$\Theta=\Phi=0^{\circ}$ and the paraxial condition that the angular spreading is small($\cos\alpha\approx1$), Eq. (1) in main text reduces to

$\tilde{\psi}(k,\alpha)=w_{0}^{|l|}\left[k_{x}+i\mathrm{sgn}(l)k_{y}\right]^{|l|}\exp\left(-\frac{w_{s}^{2}}{4}k^{2}\sin^{2}\alpha\right)\exp\left[-\frac{w_{t}^{2}}{4}\left(k-k_{0}\right)^{2}\right].$ (S1)

where

$w_{0}^{|l|}\left[k_{x}+i\mathrm{sgn}(l)k_{y}\right]^{|l|}=\left(k_{0}\sin\alpha\right)^{|l|}\exp\left(il\beta\right).$ (S2)

Now, we consider the transition from a localized spatiotemporal pulse to a continuous-wave beam. When the longitudinal pulse length extends to infinity ($w_{t}\to\infty$), the wave packet stretches indefinitely along the propagation axis *z* - *ct*. According to the mathematical limit of the Gaussian distribution, the normalized longitudinal frequency spectrum converges to the Dirac delta function

$\lim_{w_{t}\to\infty} \exp\left[-\frac{w_{t}^{2}}{4}\left(k-k_{0}\right)^{2}\right]=\delta\left(k-k_{0}\right)$ (S3)

In the polychromatic GLMT, the time-domain incident electromagnetic fields are evaluated via frequency integration over the wave number$k_{0}$. By applying the sifting property of the Dirac delta function,

$\int_{-\infty}^{+\infty} f\left(k\right)\delta\left(k-k_{0}\right)\text{d}k=f\left(k_{0}\right),$ (S4)

the polychromatic field integration analytically reduces to a single monochromatic frequency slice, evaluated precisely at the central wave number$k_{0}$, as

$\mathbf{E}^{\text{inc}}(\mathbf{r},t)=\lim_{w_{t}\to\infty} \int_{-\infty}^{+\infty} \widetilde{\mathbf{E}}^{\text{inc}}\left(\mathbf{r},k_{0}\right)\exp\left(-ikct\right)dk=\widetilde{\mathbf{E}}^{\text{inc}}\left(\mathbf{r},k_{0}\right)\exp\left(-ik_{0}ct\right),$ (S5)

and the same goes for the magnetic field. Then using the integral formula

$\int_{0}^{2\pi} \exp\left[i\left(|l|\pm1-m\right)\beta\right]d\beta=2\pi\delta_{m,|l|\pm1},$ (S6)

the BSCs in Eq. (16) in main text can be reduced to

$\left(\begin{matrix} a_{mn} \\ ib_{mn} \end{matrix}\right)=\frac{k_{0}^{2}}{2(2n+1)}\int_{0}^{\pi/2} \sin2\alpha\cos^{2}\frac{\alpha}{2}\exp\left(-\frac{k_{0}^{2}w_{s}^{2}}{4}\sin^{2}\alpha\right)\left(-\frac{k_{0}w_{s}}{4}\sin^{2}\alpha\right)^{|l|}\left[\begin{matrix} F_{mn}^{1}(\alpha)\delta_{m,|l|-1} \\ \pm F_{mn}^{2}(\alpha)\delta_{m,|l|+1} \end{matrix}\right],$ (S7)

which is consistent to the form in our previous work [S1].

**S2: Elimination of Extrinsic Chirality and Scattering Asymmetry via Unpolarized Illumination and Azimuthal‑Angle Compensation**

Before simulating the intrinsic VD of STOV, it is imperative to eliminate the interference contributions originating from the asymmetric optical background. Even when a chiral Mie sphere is strictly centered on the beam axis, significant geometric interference persists. Because of the finite volume of the Mie particle, it interacts with the spatially extended lobes of the STOV. The coupling between the linear polarization of the incident beam and the strong transverse OAM phase gradient generates a highly asymmetric distribution of transverse SAM. Integrating this asymmetric SAM over the macroscopic volume of the particle yields the “extrinsic chirality” , which produces a false chiral signal that easily masks the weak intrinsic response, as shown in Fig. S1(a). To eradicate such extrinsic interference, we employ an unpolarized STOV that is modeled as the incoherent superposition of two orthogonal linear polarization states (e.g., *x*- and *y*-polarized). Since the extrinsic SAM generated by orthogonal polarizations exhibits strictly opposite signs across the spatial profile, their incoherent sum perfectly cancels out the net extrinsic chiral background over the particle's volume:$\left(\mathbf{S}_{x}+\mathbf{S}_{y}\right)/2\equiv0$. Consequently, unpolarized excitation effectively suppresses the polarization-induced false chiral signal, ensuring a symmetric excitation environment.

**Fig. S1.** Scattering intensity of an STOV with orientation angle$(\Theta=30^{\circ},\Phi=30^{\circ})$ by a non-chiral particle. (a)The STOV is *x*-polarized. (b)The observation azimuthal angle is fixed. (c)Scattering intensity under unpolarized illumination and azimuthal observation angle compensation.

However, only eliminating the polarization-induced extrinsic chirality alone is insufficient. The pure transverse OAM of an STOV dictates that reversing the topological charge from +*l* to -*l* is physically equivalent to a spatial mirror inversion of the entire wavepacket across the OAM azimuthal plane$\Phi$. Although the particle is located at the symmetric origin, the far-field scattering lobes tilt asymmetrically corresponding to the mirrored incident wavepacket. If the differential scattering intensity is measured at a fixed observation azimuthal angle, the detector will capture completely different portions of the scattering pattern (). This intensity mismatch yields a massive non-chiral scattering background that artificially generates VD, as depicted in Fig. S1(b). To restore the parity symmetry of the background scattering field, we introduce a strict geometric angle compensation protocol. When calculating or measuring the response for -*l*, the far-field azimuthal observation angle must be correspondingly mirrored to. Under this specific coordinate transformation, the observer follows the spatial inversion of the optical field, allowing the non-chiral optical background to be perfectly matched ().

Based on unpolarized illumination with geometric observation angle compensation, we rigorously decouple the non-chiral background and extrinsic interference from the total scattering power. The resulting VD is therefore fully purified, thereby establishing a strict proportionality to the intrinsic local optical chirality, as presented in Fig. S1(c).

**S3. Correlation between Local Optical Chirality Density and Vortical Differential Scattering from a Multipolar Perspective**

In the discussion of Fig. 4 in the main text, we observed that the VD of the STOV exhibits a high degree of positive correlation with the global integral of the local optical chirality density over the particle volume. To rigorously unveil the physical mechanism underlying this phenomenon, this section provides a strict mathematical proof through multipole expansion within the framework of the GLMT. Although the actual STOV is a broadband wave packet, our mechanistic analysis rigorously focuses on the central wave number$k_{0}$. This approach is theoretically justified because the pulse energy is dominantly concentrated around$k_{0}$. Within this effective spectral bandwidth, the core electrodynamic behavior vary smoothly without qualitative phase transitions. Consequently, for arbitrarily orientations of  OAM, the optical phenomena at$k_{0}$ effectively summarize the collective behavior of the entire wave packet, providing an intuitive and rigorous demonstration of the physical mechanisms.

For a broadband incident STOV pulse with a central wave number$k_{0}$, the local optical chirality density$C\left(\mathbf{r},k_{0}\right)$ at the monochromatic slice can be strictly defined by

$\tilde{C}\left(\mathbf{r},k_{0}\right)=\frac{\varepsilon_{0}k_{0}c}{2}\mathrm{Im}\left[\tilde{\mathbf{E}}^{\text{inc}}\left(\mathbf{r},k_{0}\right)\cdot\tilde{\mathbf{H}}^{\text{inc}}\left(\mathbf{r},k_{0}\right)\right],$ (S8)

in which the$\tilde{\mathbf{E}}^{\text{inc}}\left(\mathbf{r},k_{0}\right)$ and$\tilde{\mathbf{H}}^{\text{inc}}\left(\mathbf{r},k_{0}\right)$ are the monochromatic electromagnetic field components at$k_{0}$ mentioned in the main text (Eqs. (9) and (10)). The total integrated optical chirality$K_{\text{vol}}$ induced by the pulse within the volume *V* of a chiral particle with radius$r_{p}$ can be expressed as:

$K_{\text{vol}}\left(k_{0}\right)=\int_{V} \tilde{C}\left(\mathbf{r},k_{0}\right)\text{d}^{3}\mathbf{r}.$ (S9)

Substituting Eq.(S8) into Eq.(S9) and utilizing the orthogonality relations of the VSWFs in the spherical coordinate system

$\int_{V} M_{nm}^{(1)}\cdot M_{n'm'}^{(1)}\text{d}^{3}\mathbf{r}=\delta_{nn'}\delta_{mm'},\int_{V} N_{nm}^{(1)}\cdot N_{n'm'}^{(1)}\text{d}^{3}\mathbf{r}=\delta_{nn'}\delta_{mm'},\int_{V} M_{nm}^{(1)}\cdot N_{n'm'}^{(1)}\text{d}^{3}\mathbf{r}\equiv0,$ (S10)

the total volume integrated chirality density is exclusively determined by the cross-interference terms between the electric and magnetic multipoles:

$K_{\text{vol}}\left(k_{0}\right)\propto\sum_{n=1}^{\infty} \Gamma_{n}\cdot S_{n}^{\text{inc}}=\sum_{n=1}^{\infty} \Gamma_{n}\cdot\left\{\sum_{m=-n}^{n} \mathrm{Im}\left[a_{nm}\left(k_{0}\right)b_{nm}^{*}\left(k_{0}\right)\right]\right\},$ (S11)

where$\Gamma_{n}=(2n+1)/(n^{2}+n)$ and$S_{nm}^{\text{inc}}$ is the “asymmetric excitation strength”, which characterizes the multipolar chiral excitation source term.

In the main text (Eqs. (27) and (28)), the far-field scattered electric components are expressed with angular functions$\tilde{\pi}_{n}^{m}\left(\theta\right)=\tilde{P}_{n}^{m}\left(\cos\theta\right)$ and$\tilde{\tau}_{n}^{m}\left(\theta\right)$ to depict the angle-resolved scattering patterns. However, to evaluate the total differential scattering intensity$I_{\text{sca}}$, one must integrate the scattering intensity over the entire$4\pi$ solid angle. By virtue of the rigorous orthogonality of the angular functions

$\int_{0}^{\pi} \left[\pi_{n}^{m}\left(\theta\right)\pi_{n'}^{m'}\left(\theta\right)+\tau_{n}^{m}\left(\theta\right)\tau_{n'}^{m'}\left(\theta\right)\right]\sin\theta\text{d}\theta=\frac{2n\left(n+1\right)}{2n+1}\delta_{nn'}\delta_{mm'},$ (S12)

the complex angular dependencies are analytically integrated out. Consequently, the full-space integral of scattering intensity can be reduced to the sum of the modulus squared of the scattering coefficients$a_{nm}^{s}\left(k\right)$ and$b_{nm}^{s}\left(k\right)$ as

 (S13)

In the expanding series of the modulus squared terms$\left|a_{nm}^{s}\right|^{2}+\left|b_{nm}^{s}\right|^{2}$, apart from the symmetric self-multiplication terms, the cross-polarization inevitably generates asymmetric interference terms $2\mathrm{Re}\left(-id_{n}e_{n}^{*}a_{mn}b_{mn}^{*}\right)+2\mathrm{Re}\left(ic_{n}e_{n}^{*}b_{mn}a_{mn}^{*}\right)$. After the complex derivation, Eq. (S6) can be expressed as

 (S14)

**Fig. S2.** Multipolar analysis of (a) the asymmetric excitation strength$S_{nm}^{\text{inc}}$ for an STOV with orientation angle$(\Theta,\Phi)$ and (b) the resonant amplification weight coefficient$W_{n}$.

where$W_{n}=(2n+1)\mathrm{Re}[(d_{n}+c_{n})e_{n}^{*}]/(n^{2}+n)$ represents the resonant amplification weight coefficient of the *n*-th order Mie multipole. Comparing Eq. (S11) and Eq. (S14) reveals that the local chirality density integral$K_{\text{vol}}\left(k_{0}\right)$ and the VDS share the same fundamental driving source—the asymmetric excitation strength$S_{nm}^{\text{inc}}$. This elucidates why both quantities exhibit a highly consistent evolutionary trend (positive correlation).

As illustrated in Fig. S2, when the orbital angular momentum (OAM) orientation angle is purely transverse$(\Theta=90^{\circ},\Phi=90^{\circ})$, the asymmetric excitation strength strictly vanishes ($S_{n}^{\text{inc}}\equiv0$). The total volume chirality$K_{\text{vol}}\left(k_{0}\right)$ will inevitably collapse to zero and the vortical differential scattering (VDS) cannot be excited. Meanwhile, for the Mie spherical particle with radius$r_{p}=1.0\lambda_{0}$, the dominant contributions to the VDS originate mainly from the lower-order Mie multipoles, specifically within the range of *n* = 1 to 6. Within this regime, the asymmetric excitation strength$S_{n}^{\text{inc}}$ at an OAM orientation angle of$(\Theta=30^{\circ},\Phi=30^{\circ})$ is notably higher than that at$(\Theta=0^{\circ},\Phi=0^{\circ})$. As a result, this enhanced multipolar cross-interference effectively drives a significantly larger VDS compared to the purely longitudinal OAM incidence. This derivation in the multipolar dimension not only confirms the validity of the phenomena presented in Figure 4 but also provides a universal theoretical benchmark for the interaction between complex structured optical fields and chiral matter.

**S4. Analysis of Energy Flow Vortex Collapse and Topological Torque**

In Fig. 5 of the main text, we visually demonstrate the evolution of the spatial Poynting vector streamlines with respect to the varying OAM orientation angle$(\Theta,\Phi)$. At the monochromatic central wave number slice$k_{0}$, the time-averaged Poynting vector of the optical field is defined as

$\left\langle\tilde{\mathbf{S}}\left(\mathbf{r},k_{0}\right)\right\rangle=\frac{1}{2}\mathrm{Re}\left[\tilde{\mathbf{E}}\left(\mathbf{r},k_{0}\right)\times\tilde{\mathbf{H}}^{*}\left(\mathbf{r},k_{0}\right)\right].$ (S15)

Under the paraxial approximation, the transverse energy flow$(\tilde{S}_{x},\tilde{S}_{y})$ is predominantly governed by the spatial phase gradient of the scalar electric field. For an optical field carrying arbitrary topological phase of the form$\tilde{\mathbf{E}}=\left|\tilde{\mathbf{E}}\right|\exp\left(i\psi\right)$ in cylindrical coordinates, its transverse energy flow can be approxi- mately expressed as

$\mathbf{S}_{\perp}(\mathbf{r})\approx\frac{\varepsilon_{0}c}{2}|\tilde{\mathbf{E}}|^{2}\nabla_{\perp}\psi,$ (S16)

with

 (S17)

When the beam carries OAM with a longitudinal component $(\Theta\neq90^{\circ},\Phi\neq90^{\circ})$, the phase gradient$\nabla_{\perp}\psi$ has non-zero azimuthal components, and the energy flow distribution features strong circulation. This rotational energy flow field swirling around the propagation axis exerts a net electrodynamic topological torque within the volume *V* occupied by the particle. The torque density is defined as the cross product of the position vector and the transverse energy flow

$\mathbf{\tau}_{\text{orb }}\propto\oint_{V} \left(\mathbf{r}\times\mathbf{S}_{\perp}\right)\text{d}^{3}\mathbf{r}\neq0.$ (S18)

Under the paraxial approximation, for a linearly polarized vortex beam, the OAM-induced optical chirality arises exclusively from the longitudinal field components induced by transverse spatial gradients. Analytically, the local optical chirality density can be mapped onto the radial gradient of the axial topological torque density

$\tilde{C}\left(\mathbf{r},k_{0}\right)\propto\frac{k_{0}}{r}\frac{\partial\tau_{\text{orb}}^{z}}{\partial r}.$ (S19)

As derived in Section S2, the VDS is fundamentally governed by the volume integral of the local optical chirality. Therefore, for the case of$(\Theta\neq90^{\circ},\Phi\neq90^{\circ})$, Eq. (S18) yields a non-zero torque component along the *z*-axis. It is precisely this “spatial twisting torque”, acting like a turbine, that strongly couples with the cross-polarized eigenmodes inside the chiral particle. Reversing the topological charge directly flips the direction of this torque, thereby generating the highly pronounced VDS observed in Fig. 3.

However, when the beam evolves into a STOV with OAM orientation$(\Theta=90^{\circ},\Phi=90^{\circ})$, the spatiotemporal symmetry of the system undergoes a fundamental transition. On the *x-y* plane with the central wave number$k_{0}$, $\nabla_{\perp}\psi\equiv0$. This implies that the phase gradient of the spatial wavefront at the central frequency completely loses its circulating characteristic component along the azimuthal direction. Consequently, the spatial energy flow vortex undergoes a complete collapse, with the streamlines degenerating from closed helical vortices into divergent or symmetric linear structures. This reveals that the macroscopic electrodynamic twisting torque acting on the particle strictly vanishes. Although this degenerate localized field can still provide extremely high energy density (exciting intense total Mie scattering intensity), it completely loses the topological driving force capable of breaking the symmetry between left- and right-handed molecular/structural responses. In the absence of this requisite torque, asymmetric chiral multipolar interference cannot be established within the particle, leading directly to the extinction of the vortical differential scattering response under the transverse OAM configuration.

**Reference:**

S1 [Y. S](https://www.x-mol.com/paper/1248467243967479808)hi, [Z. C](https://www.x-mol.com/paper/1248467243967479808)ui, Z. Liu, S. Ren, and F. Wu, “Description and reconstruction of typical structured light beams with vector spherical wave functions,” *Appl. Opt.*, **63**, 2392-2403 (2024).
